# The knee kinematic patterns and associated factors in healthy Thai adults

**DOI:** 10.1186/s12891-023-07081-7

**Published:** 2023-12-05

**Authors:** Tanyaporn Patathong, Krongkaew Klaewkasikum, Chanika Angsnuntsukh, Thira Woratanarat, Chusak Kijkunasathian, Jongsook Sanguantrakul, Patarawan Woratanarat

**Affiliations:** 1 Department of Orthopaedics, Faculty of Medicine Ramathibodi Hospital, Mahidol University, 270 Rama VI Road, Ratchathewi, Bangkok, 10400 Thailand; 2 Department of Preventive and Social Medicine, Faculty of Medicine, Chulalongkorn University, Bangkok, 10330 Thailand; 3 National Electronics and Computer Technology Center, National Science and Technology Development Agency, Pathumthani, 12120 Thailand

**Keywords:** Gait cycle, Motion analysis, Tibial rotation, Side difference, Walking

## Abstract

**Background:**

Reference values for normal knee kinematics were limited in Asian population and were influenced by race and other factors. This study was aimed to establish the reference values and identify the factors associated with knee kinematics in healthy Thai adults, aged 18–40 years.

**Methods:**

A retrospective cohort study was conducted between 2016 and 2020. Healthy Thai adults aged 18–40 years old with body mass index (BMI) between 18.5 and 24.9 kg/m^2^ were included. All eligible participants were attached with reflective markers. Their walking was captured by 8-digital cameras, and assessed by motion analysis software. The primary outcomes were average knee kinematic data (degrees) in three dimensional planes as valgus-varus, flexion-extension, and internal-external rotation. Paired t-test and multiple linear regression were applied to compare the outcomes and to determine their associated factors.

**Results:**

Ninety-eight participants (60 females and 38 males) were included with mean age 28.5 ± 5.4 years, and BMI 21.1 ± 2.0 kg/m^2^. Knee kinematics showed slight adduction during the swing phase, flexion during the stance phase, and obvious external rotation throughout the gait cycle, with a peak of 30–31 degrees during mid-swing. Right knee was significantly more adducted, flexed and externally rotated than the left side, particularly at mid-stance (*P* = 0.047, 0.017, and < 0.001, respectively). Females had more knee abduction, flexion and external rotation than males. Age, sex, and BMI were significantly correlated with knee abduction at terminal stance (correlation coefficient − 0.12, 95% confidence interval (CI) -0.23, -0.01; -1.37, 95%CI -2.54, -0.20; and − 0.32, 95%CI -0.61, -0.39, respectively), and rotation at mid-swing (correlation coefficient − 0.36, 95%CI -0.69, -0.02; -7.37, 95%CI -10.82, -3.92; and 0.89, 95%CI 0.01, 1.78, respectively).

**Conclusion:**

Knee kinematics demonstrates external tibial rotation throughout the gait cycle, significant side differences, and are associated with age, sex, and BMI. Reference values from this study will be useful for functional gait assessment in healthy Thais. However, further comprehensive knee kinetic study including spatio-temporal parameter is recommended.

**Supplementary Information:**

The online version contains supplementary material available at 10.1186/s12891-023-07081-7.

## Introduction

The knee is the largest synovial hinge joint, which primarily allows flexion and extension as well as angulation, rotation, and translation to facilitate walking and perform activities [1]. The natural knee weight-bearing state during walking cannot be determined by simple physical examination and radiographs [2]. Three-dimensional (3D) motion analysis is widely used to evaluate knee movements in sagittal (flexion-extension), frontal (valgus or abduction-varus or adduction), and transverse planes (internal-external rotation) [3]. This method requires values from normal adults to help identify an abnormal motion.

Most knee kinematic references were from European and Scandinavian populations [3–8]. Although knee kinematics has been recently studied among Asians, only few studies were involved with normal adults [9, 10]. The data from Thailand were particularly limited to only the sagittal plane of the initial gait cycle [11]. Moreover, spatiotemporal, kinematic and kinetic data between Asian and European studies were significantly diverse [10], and influenced by several factors [12] such as age [7, 11, 13], sex [6, 7, 9, 11, 14–17], body mass index (BMI) [18, 19], side [20], and ethnicity [10]. Middle-aged people and elderly developed neuromuscular physiology or behavioral changes of knee kinematics [13]. Females tend to have more laxity, and less stiffness in knee external rotation than males [16].

The purpose of this study was to evaluate three-dimensional knee kinematics in healthy Thai adults 18–40 years old, and to identify associated factors. Establishing normal gait data from this commonly used age range [10, 21, 22], this study’s findings would be beneficial for treatment and follow-up plans for patients with knee problems. The data could be useful for healthy adults with similar anthropometry and chronological age.

## Materials and methods

After the Institutional Review Board approval (COA. MURA2021/50), a retrospective cohort study was conducted at a Gait Laboratory in a university hospital, Bangkok, Thailand during 2016–2020.

### Participants

Healthy Thai adults aged 18–40 years who had BMI between 18.5 and 24.9 kg/m^2^ were included. This age range was specified in order to omit the toe off reduction effect in adults age over 40 years old [11, 22]. Subjects with high BMI were not involved due to possible abnormal motion from excess load [23]. The exclusion criteria were conditions which affected the normal gait pattern, i.e., having previous history of musculoskeletal and neurological disorders related to lower extremities within 6 months [24]. Medical records and/or physical examination were verified by two physiotherapists where appropriate.

### Motion analysis

**Fig. 1:**
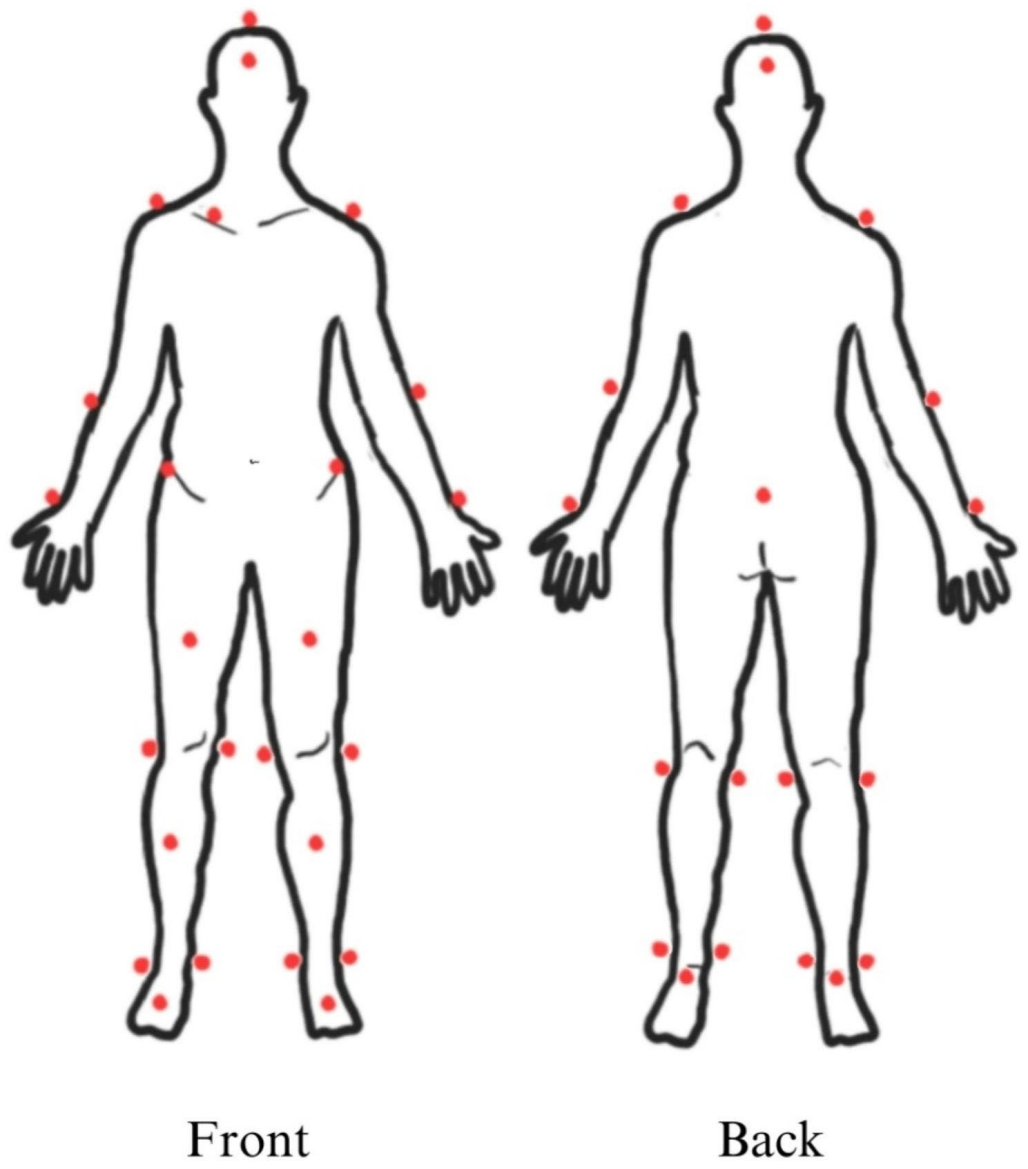
The modified Helen Hayes technique

Twenty-nine reflective makers were attached over participants’ bony landmarks in accordance with the modified Helen Hayes technique by two well-trained gait lab physiotherapists (Fig. 1). All participants walked barefoot along an 8-meter straight walkway for 8–10 rounds with their preferred walking speed to identify their intimate walking simultaneous with all reflective markers detection. Walking data and the gait cycle were captured by 8 digital motion cameras (Motion Analysis Corp, CA, USA). The gait parameters and raw kinematic data were analyzed by Cortex-64 6.2.0.1714 and Orthotrak 6.6.1 software.

### Data collection

Demographic data including age (year), sex, weight (kg), height (cm), BMI (kg/m^2^), gait velocity (m/s), and stride length (m) were collected from interview, physical examination, and gait parameters from Motion analysis software, respectively. Clinical outcomes were knee kinematics (degrees) retrieved from average data of each participant’s best 6 right and 6 left gait cycles (2 cycles of 3 gait trials). One gait cycle was defined as complete heel strike to heel strike of the same foot. Three-dimensional planes were reported as sagittal (flexion-extension); frontal (valgus or abduction–varus or adduction), and transverse planes (internal-external rotation). By using Cortex and Orthotrak software, each plane was captured at 0–12%, 12–31%, 31–50%, and 74–87% of gait cycle (0-100%) for heel strike, mid-stance, terminal stance and mid-swing, respectively [25]. At stance phase, pre-swing (51–60% of gait cycle) was not included because it was close to terminal stance with double limb supports. At swing phase, mid-swing which represented maximum knee flexion was selected.

### Statistical analysis

The sample size was 98 people based on alpha error 0.05, beta error 0.2, the mean sagittal knee angle 3.26 ± 8.46 degrees at heel strike from the study in Thais [11], and the mean sagittal knee angle 5.7 degrees from the Australian study [2]. This estimation covered the sample size calculation from China [9, 10], and Korea [1]. Baseline characteristics and knee kinematic data were presented as mean (standard deviation) for continuous data, and frequency (percentage) for categorical data. Kolmogorov-Smirnov test and paired t-test were used to test the normality of the data and to compare the difference, respectively. Multiple linear regression was performed to explore the association between factors and outcomes (correlation coefficient and 95% confidence interval (CI). STATA 16.0 (StataCorp, College Station, Texas, USA) was used for sample size calculation, and all analyses. The statistical significance was set at *P* < 0.05.

Statistical parametric mapping (SPM) was applied for full-time series of the angle assessment. The knee kinematics were compared between sides using two-tailed paired t-test, and between the study samples (healthy Thai adults) and the reference values supplied by the motion analysis program using two-tailed unpaired t-test. The analysis was computed by MATLAB (R2020b, The MathWorks Inc) with open-source code (M.0.4.8, www.spm1d.org). The significance level was set at alpha error = 0.05.

## Results

### Characteristics of participants

Demographic data of participants were presented in Table 1. Among the total of 98 participants, 60 (61.2%) were women and 38 (38.8%) were men. The mean age was 28.5 ± 5.4 years with the BMI of 21.1 ± 2.0 kg/m^2^, and gait velocity at 1.0 ± 0.1 m/s.

**Table 1:** Demographic characteristics of participants

Characteristics	Value (n = 98)
Age (year), mean (SD)	28.5 (5.7)
Male/Female, n (%)	38 (38.8) / 60 (61.2)
Weight (kg), mean (SD)	58.3 (9.4)
Height (cm), mean (SD)	165.7 (7.8)
BMI (kg/m^2^), mean (SD)	21.1 (2.0)
Gait velocity (m/s), mean (SD)	1.1 (0.1)
Stride length (m), mean (SD)	1.2 (0.1)

Abbreviation - SD = standard deviation, BMI = body mass index

### Knee kinematics data

Knee kinematics data were reported in 4 phases of the gait cycle, including heel strike, mid-stance, terminal stance, and mid-swing. Reference data of right and left knee kinematics during walking were shown in Table 2.

**Table 2:** Right and left knee kinematics of healthy subjects

Knee kinematics (degree)	Right knee		Left knee		P-value
	Mean	SD	Mean	SD	
Varus-valgus					
Heel strike	0.69	2.98	0.44	3.27	0.635
Mid-stance	0.84	3.16	0.33	3.13	0.047*
Terminal stance	-0.24	3.17	-0.41	3.09	0.659
Mid-swing	4.98	5.98	3.35	5.63	< 0.001*
**Flexion-extension**					
Heel strike	10.49	4.27	10.16	4.49	0.346
Mid-stance	16.33	5.09	15.34	5.18	0.017*
Terminal stance	14.85	4.09	14.28	3.99	0.071
Mid-swing	63.58	4.29	63.78	4.62	0.577
**Internal-external rotation**					
Heel strike	-28.72	10.59	-25.53	9.87	0.002*
Mid-stance	-22.95	10.28	-19.29	9.61	< 0.001*
Terminal stance	-19.49	9.89	-15.97	9.26	< 0.001*
Mid-swing	-31.78	10.71	-30.61	9.37	0.195

+ = knee varus, flexion, and internal rotation; - = knee valgus, extension, and external rotation; *significant (*P* < 0.05), SD = standard deviation

### The frontal plane

**Fig. 2:**
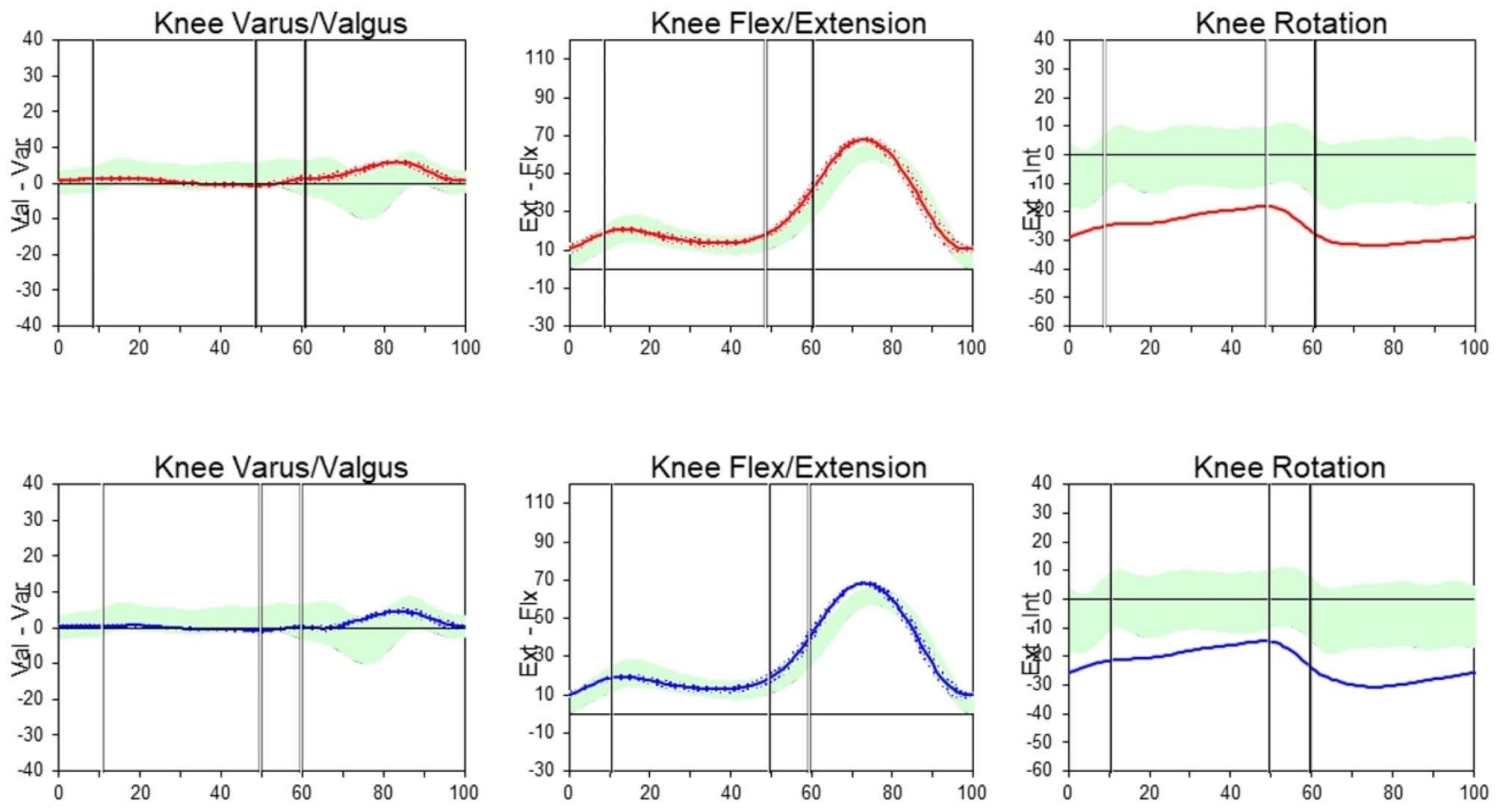
The average kinematics pattern of the right knee (upper row) and the left knee (lower row). The red lines define the average right knee kinematics of Thai adults. The blue lines define the average left knee kinematics of Thai adults. The green area are normal values supplies of knee kinematics from the Orthotrak software (Orthotrak, Motion Analysis Corp, CA, USA)

Average varus-valgus kinematic pattern of the right and the left knees during a gait cycle was presented, (Fig. 2). The curve demonstrated knee adduction (varus) at heel strike and mid-stance (< 1 degree). The knee gradually moved to neutral alignment during terminal stance, and then reached knee abduction (< 1 degree). Finally, the curve returned to adduction at the swing phase, and rose to the peak knee adduction (3–4 degrees) during mid-swing. There were significant side differences at mid-stance (*P* = 0.047) and mid-swing (*P* < 0.001).

### The sagittal plane

Regarding flexion-extension motion of both knees (Fig. 2), their curves followed a similar pattern. The knee was initially 10-degree flexed at heel strike, and slightly increased to 15–16 degrees at mid-stance. The knee flexion then decreased to 14 degrees during terminal stance. The peak knee flexion was 63 degrees during mid-swing. A significant difference between sides was detected at mid-stance (*P* = 0.017).

### The transverse plane

Bilateral internal-external tibial rotation followed a similar pattern (Fig. 2). The curve started at 25–28 degrees of external rotation, and slightly decreased during mid-stance and terminal stance. The peak external rotation was 30–31 degrees at mid-swing. Side differences were observed at heel strike (*P* = 0.002), mid-stance (*P* < 0.001), and terminal stance (*P* < 0.001).

### Statistical parametric mapping (SPM)

**Fig. 3:**
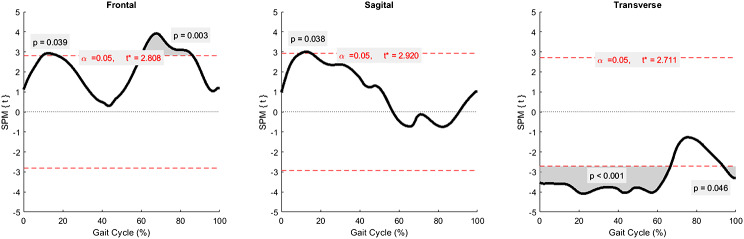
Statistical parametric mapping (SPM) compared knee kinematics between the right and left sides. Shaded areas determined significant differences at points of time

SPM analysis of knee kinematics reported significant side inequality (Fig. 3). Right knee kinematics were more varus at mid-stance (*P* = 0.039) and mid-swing (*P* = 0.003), more flexion at mid-stance (*P* = 0.038), and more external rotation at heel contact through terminal stance (*P* < 0.001) as well as the swing phase (*P* = 0.046) than the left side.

**Fig. 4:**
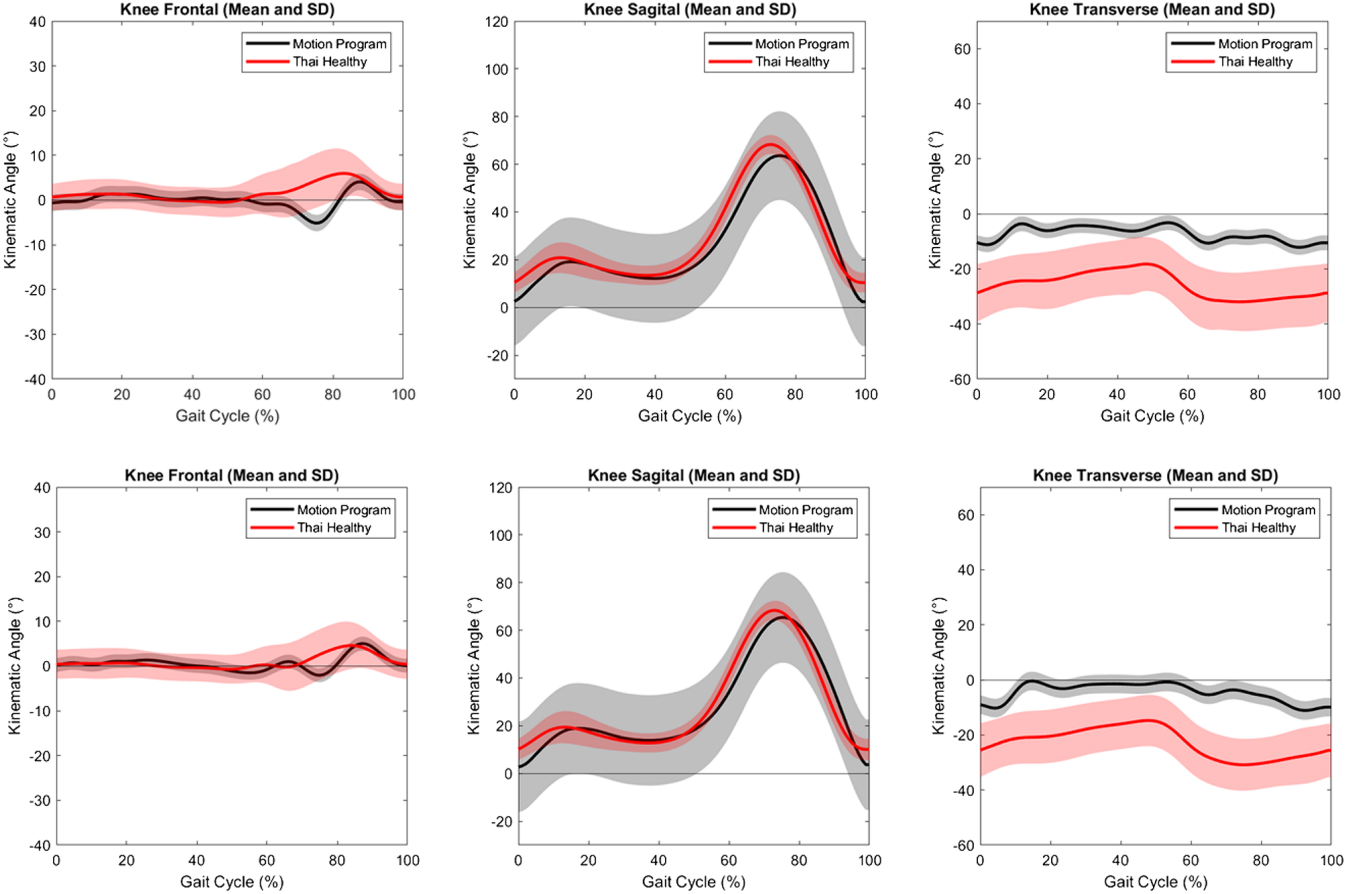
The kinematic pattern of the right knee (upper row) and left knee (lower row). The average (lines) and standard deviation (area) of knee kinematics; red color for healthy Thai adults and grey color for normal knee kinematics were supplied by the Orthotrak software (Orthotrak, Motion Analysis Corp, CA, USA)

**Fig. 5:**
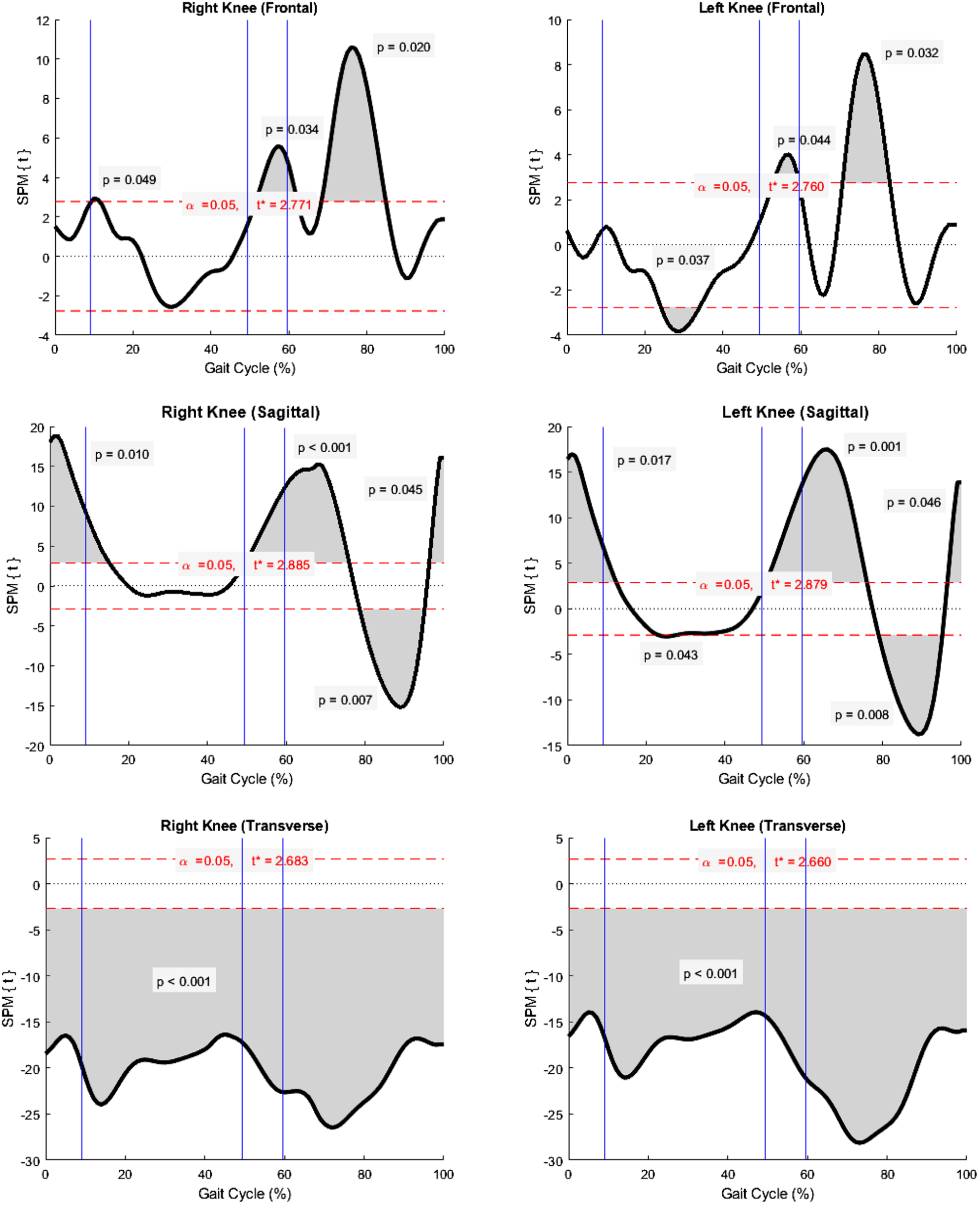
Statistical parametric mapping (SPM) compared knee kinematics between healthy Thai adults and normal knee kinematics supplied by the Orthotrak software (Orthotrak, Motion Analysis Corp, CA, USA) of each side. Shaded areas determined significant differences at points of time

Knee kinematics of healthy Thai adults and normal values supplied by Orthotrak Software of Motion analysis program were plotted for the right and the left knees (Fig. 4). SPM analysis demonstrated significant differences between these two databases (Fig. 5). Data from healthy Thai adults was more varus (mid-stance, terminal swing, and mid-swing) with more flexion and external rotation (entire gait cycle) than that of Orthotrak Software.

### Correlation analysis

#### Age correlation

Advanced age was correlated with decremental knee varus and internal rotation (Table 3). In the frontal plane, every incremental 1 year of age demonstrated less knee varus by 0.12 degrees at terminal stance (coefficient − 0.12, 95%CI -0.23, -0.01; *P* = 0.027). In the transverse plane, every incremental 1 year of age indicated less knee internal rotation by 0.36 degrees at mid-swing (coefficient − 0.36, 95%CI -0.69, -0.02; *P* = 0.037).

**Table 3:** The correlation coefficient between knee kinematics and age gender, and BMI

Knee kinematics (degree)	Age (years)			Female sex			BMI (kg/m^2^)		
	coefficient	95% CI	P-value	coefficient	95%CI	P-value	coefficient	95%CI	P-value
**Varus-valgus**									
Heel strike	-0.08	-0.19, 0.03	0.140	-1.09	-2.29, 0.09	0.072	-0.33	-0.62, -0.05	0.023*
Mid-stance	-0.10	-0.21, 0.01	0.063	-0.59	-1.79, 0.61	0.332	-0.46	-0.74, -0.19	0.001*
Terminal stance	-0.12	-0.23, -0.01	0.027*	-1.37	-2.54, -0.20	0.022*	-0.32	-0.61, -0.39	0.026*
Mid-swing	-0.13	-0.32, 0.07	0.204	-1.27	-3.42, 0.87	0.241	-0.29	-0.80, 0.23	0.276
**Flexion-extension**									
Heel strike	0.03	-0.12, 0.18	0.698	0.78	-0.88, 2.45	0.353	-0.08	-0.48, 0.32	0.693
Mid-stance	0.02	-0.16, 0.19	0.847	-1.02	-2.96, 0.92	0.299	0.05	-0.43, 0.52	0.850
Terminal stance	0.01	-0.13, 0.15	0.898	1.19	-0.32, 2.71	0.120	-0.19	-0.57, 0.17	0.288
Mid-swing	0.04	-0.12, 0.19	0.645	2.80	1.20, 4.39	0.001*	-0.19	-0.61, 0.21	0.333
**Internal-external rotation**									
Heel strike	-0.22	-0.56, 0.13	0.209	-4.93	-8.60, -1.25	0.009*	0.95	0.05, 1.85	0.040*
Mid-stance	-0.16	-0.49, 0.17	0.345	-4.32	-7.88, -0.76	0.018*	1.13	0.28, 1.99	0.010*
Terminal stance	-0.12	-0.44, 0.19	0.444	-3.38	-6.82, 0.06	0.054	0.95	0.12, 1.77	0.025*
Mid-swing	-0.36	-0.69, -0.02	0.037*	-7.37	-10.82, -3.92	< 0.001*	0.89	0.01, 1.78	0.049*

+ = knee varus, flexion, and internal rotation; - = knee valgus, extension, and external rotation;

*significant (*P* < 0.05), CI = confidence interval

### Sex correlation

Females demonstrated less knee varus, and internal rotation, but more knee flexion when compared to males (Table 3). In the frontal plane, females showed 1.37 degrees less knee varus at terminal stance than males (coefficient − 1.37, 95%CI -2.54, -0.20; P = 0.022). In the sagittal plane, females increased knee flexion by 2.8 degrees at mid-swing when compared to males (coefficient 2.80, 95%CI 1.20, 4.39; P = 0.001). For the transverse plane when compared to males, females decreased knee internal rotation by 4.93 degrees at heel strike (coefficient − 4.93, 95%CI -8.60, -1.25; P = 0.009), 4.32 degrees at mid-stance (coefficient − 4.32, 95%CI -7.88, -0.76; P = 0.018), and 7.37 degrees at mid-swing (coefficient − 7.37, 95%CI -10.82, -3.92; P < 0.001).

### BMI correlation

Incremental BMI was correlated with decreased knee varus and increased knee internal rotation (Table 3). In the frontal plane, every incremental 1 kg/m^2^ of BMI reduced knee varus by 0.33 degrees at heel strike (coefficient − 0.33, 95%CI -0.62, -0.05; P = 0.023), 0.46 degrees at mid-stance (coefficient − 0.46, 95%CI -0.74, -0.19; P < 0.001), and 0.32 degrees at terminal stance (coefficient − 0.32, 95%CI -0.61, -0.39; P = 0.026). For the transverse plane, every incremental 1 kg/m^2^ of BMI increased knee internal rotation by 0.95 degrees at heel strike (coefficient 0.95, 95%CI 0.05, 1.85; P = 0.040), 1.13 degrees at mid-stance (coefficient 1.13, 95%CI 0.28, 1.99; P = 0.010), 0.95 degrees at terminal stance (coefficient 0.95, 95%CI 0.12, 1.77; P = 0.025) and 0.89 degrees at mid-swing (coefficient 0.89, 95%CI 0.01, 1.78; P = 0.049).

### Regression models

The predicted regression models revealed age, sex, and BMI were significantly correlated with knee varus at terminal stance, and knee internal rotation at mid-swing of each side and both knees, Additional file 1: Table S1-S6. The models of both knees (Additional file 1: Table S1 and S4) were presented as the following equations.

- Knee varus at terminal stance = 14.87 − 0.1(age) -2.6(sex; 0 = male/ 1 = female) -0.6(BMI).
- Knee internal rotation at mid-swing = (-27.1) -0.3(age) -6.1(sex; 0 = male/ 1 = female) + 0.3(BMI).

## Discussion

Although knee kinematics during walking has been widely studied, there is still lack of information among the Thai population. We investigated all aspects of knee kinematic data in the three planes for four phases of gait cycle including heel strike, mid stance, terminal stance, and mid-swing. This study showed that knee kinematics was close to 0 degree during the stance phase and turned to adduction (varus) during the swing phase. Additionally, knee external rotation was found throughout the gait cycle. Age, sex, and BMI contributed to knee motion mostly in knee varus (terminal stance) and knee internal rotation (mid swing).

Knee kinematics varied with racial, cultural, and ethnic properties. Moreover, knee motion might be influenced by anatomical coordinate systems [9], hip mechanical abnormalities [26, 27], movement conditions [1], marker placement, calculation methods of knee kinematics, measurement systems, and the calibration procedure, particularly in the frontal and transverse planes [22, 28–32]. From the studies comparing gait characteristics between Asian (China and Korean) [1, 10, 21, 33] and Western adults (Australian, Canadian, Italian) [2, 6, 34], they showed similarities in motion patterns and excursions, but differences in the ranges of angles (Table 4). The range of knee kinematics of the entire gait cycle in Asians, as measured by the Opti-Knee, Motion analysis, Midas system, and Vicon were − 4.4 to 5.5 [33], -10.1 to 3.7 [1], 6.6 to 13.0 [10], and − 1.0 to 7.5 [21] degrees for abduction-adduction and − 4.4 to 9.6 [33], -5.6 to 17.4 [1], -8.2 to 3.3 [10], -6.0 to 5.0 [21] degrees for internal-external rotation, respectively. In contrast, the range of knee kinematics from the entire gait cycle among Western studies, as measured by KneeKG and Elite, were − 4.8 to 5.5 [6], -0.6 to 4 [2], and − 6.5 to 4.1 [34] degrees for abduction-adduction and − 2.8 to 3.7 [6], -7.1 to 6.1 [2], and − 5.3 to 8.4 [34] degrees for internal-external rotation, accordingly.

**Table 4:** Comparison of hip and knee kinematics between studies conducted in Asian and Western studies

Variables	This study	Liu R, 2021	Kim HY, 2015	Ryu T, 2006	Cho HS, 2004	Clément J, 2018	Mannering N, 2017	Benedetti MG, 1998
Age (years) mean ± SD (range)	28.5 ± 5.7 (18–40)	21.9 ± 2.0	Female 20.0 Male 20.0	Female 24.1 ± 1.6 Male 24.9 ± 2.0	Female 22.9 ± 4.9 Male 23.5 ± 2.7	Female 34.8 ± 12.7 Male 34.8 ± 12.1	Female 23.2 ± 3.0 Male 24.3 ± 4.0	43.0 (18.0) (20–72)
Country	Thailand	China	Korea	Korea	Korea	Canada	Australia	Italy
System	Motion Analysis (Santa Rosa, USA)	Opti-Knee (Shanghai, China)	Motion Analysis (Santa Rosa, USA)	Midas system (Woburn, USA)	Vicon 370 (Oxford, UK)	KneeKG system (Montreal, Canada)	KneeKG system (Montreal, Canada)	Elite system (Milano, Italy),
Velocity (m/s), mean ± SD	1.1 ± 0.1	N/A	Female 1.3 Male 1.3	1.1 ± 0.1	1.2 ± 0.1	1.1 ± 0.1	N/A	1.3 (0.2)
Stride length (m), mean ± SD	1.2 ± 0.1	N/A	Female 1.3 Male 1.4	1.3 ± 0.1	1.2 ± 0.1	N/A	N/A	1.4 (0.2)
Hip AD-AB (total excursion)	-6.8, 10.9 (17.7)	N/A	N/A	-14.1, -0.2 (13.9)	-4.3, 7.3 (11.6)	N/A	N/A	-5.5, 5.4 (10.9)
Hip F-E (total excursion)	-19.7, 50.3 (69.9)	N/A	N/A	-18.3, 27.4 (45.7)	-4.2, 39.0 (43.2)	N/A	N/A	-10, 29.8 (39.8)
Hip IR-ER (total excursion)	-9.8, 24.3 (34.1)	N/A	N/A	0.4, 9.8 (9.4)	-5.0, 8.0 (13.0)	N/A	N/A	-3.4, 8.5 (11.9)
Knee varus-valgus (total excursion)	-9.6, 6.9 (16.5)	-4.4, 5.5 (9.9)	-10.1, 3.7 (13.8)	6.6, 13.0 (6.7)	-1.0, 7.5 (8.5)	-4.8, 5.5 (10.3)	-0.6, 4.0 (4.6)	-6.5, 4.1 (10.6)
Knee F-E (total excursion)	0.8, 73.8 (74.6)	1.7, 62.6 (60.9)	-1.4, 62.4 (63.8)	1.4, 59.4 (58)	2.3, 59.0 (56.7)	-10.0, 56.0 (66.0)	0.7, 58.0 (57.3)	0.4, 65.7 (65.3)
Knee IR-ER (total excursion)	-50.5, 0.5 (51.0)	-4.4, 9.6 (14.0)	-5.6, 17.4 (23.0)	-8.2, 3.3 (11.5)	-6.0, 5.0 (11.0)	-2.8, 3.7 (6.5)	-7.1, 6.1 (13.2)	-5.3, 8.4 (13.7)

Knee kinematics (degrees): AD = adduction, AB = abduction, F = flexion, E = extension, IR = internal rotation, ER = external rotation, N/A = not available,

+ = adduction, flexion, internal rotation, and varus, - = abduction, extension, external rotation, and valgus

This study’s data is consistent with previous researches in terms of knee adduction-abduction [5], flexion-extension [2–9, 14, 17, 20, 35, 36], and external rotation patterns [20, 35, 37]. However, some studies otherwise reported knee abduction [20, 37], or adduction throughout the gait cycle [9]; internal rotation in the entire gait cycle [38, 39], or knee external rotation during the stance phase and turned to internal rotation during the swing phase [1, 9]. Nevertheless, it is worth noting that the knee motion of Thai adults clearly exhibited more adduction, and knee external rotation when compared to the motion analysis program (Figs. 2 and 4, and 5) and other studies [1, 2, 6, 10, 21, 33, 34], as shown in Table 4. Excessive hip adduction and internal rotation could affect knee valgus/abduction and external rotation [26, 27]. The axis of knee rotation also shifted laterally during weight bearing [1].

This study found significant side difference (Table 2; Fig. 3), especially in the transverse plane (internal-external tibia rotation) in both stance and swing phases (*P* < 0.05). The right side indicated more external rotation at heel strike, adduction-flexion-external rotation at mid-stance, external rotation at terminal stance, and adduction at mid-swing than the left one. However, dominant leg side could not be adjusted due to unavailable data from this study. The knee kinematics from Oberg et al. in Sweden did not demonstrate side-to-side significance [7], whereas the study by Ino et al. from Japan reported side inequality in adduction-abduction, especially during the swing phase [20]. Mechanical restraint of the tibial motion may affect the side difference during the stance phase, and ligament balance may play a major role for joint kinematics in the swing phase [20]. For the differences detected in Thai adults, we hypothesized that traditional Thai sitting habit, a usual right-side swipe in cross legs on the floor, may affect excessive right hip internal rotation and right knee external rotation.

Age and sex were associated with knee range of motion regarding gait cycle [7, 14, 17, 21, 40]. With wider age range (10–79 years) than this study (18–40 years), Oberg et al. [7] similarly reported that age was significantly related with minor change of knee angle at mid stance (0.5 degree/decade). However, this study also found that advanced age significantly increased knee valgus at terminal stance (0.12 degree/year), and external rotation at mid swing (0.36 degree/year).

Thai females demonstrated more external rotation at heel strike, mid-stance and mid-swing, abduction at terminal stance, and flexion-external rotation at mid-swing than males. With regards to sex, females tended to have knee extension in the stance phase and turned to be more flexion in mid swing [14]. They also had significant knee valgus/abduction throughout the gait cycle when compared to males [9, 21, 41].

BMI was directly correlated with knee abduction and internal rotation in the stance phase and internal rotation in mid-swing. The reduction of body weight was associated with decreased range of adduction-abduction [19]. On the other hand, increasing weight might contribute to knee abduction and internal rotation. Moreover, BMI affected the pattern of knee adduction and rotation moment as seen in knee osteoarthritis [18].

From the regression models (Additional file 1: Table S1), females had higher knee valgus angle than males at terminal stance which corresponded with the previous studies [9, 21, 41]. In addition, they had higher knee external rotation than males in the mid-swing phase of both sides **(Additional file 1: Table**S4**)**. Hip internal rotation could increase knee external rotation [26, 27]. Culturally, Thai females were traditionally taught to sit on the floor (with one leg tucked back to one side). This sitting position could promote hip internal rotation, and knee external rotation. Moreover, females had higher laxity in external rotation, lower stiffness [16], and also 10–20% larger range of knee rotation than males during knee flexion [42].

The strength of this study is that the knee kinematic data were objectively measured by the standard 3D-Motion gait analysis with the modified Helen Hayes marker set. This study considered age, sex and BMI associated with knee motions entire gait cycle. However, there were some limitations that the sample size might not be large enough to represent the whole population in Thailand. Inadequate number of subjects did not permit subgroup analysis by age, sex, height, weight, and BMI. The dominant side [43], hip and ankle kinematics were not taken into account. The reference values reported through the present study can only be applied for age 18–40 years old with normal BMI.

For the clinical application, healthy young Thai adults may utilize the normal gait pattern in this study as a reference. Adjustments for age, sex, and BMI by using the predicted models from this study, and side difference should also be considered.

## Conclusion

This study provides useful knee kinematic references for functional gait of healthy Thai adults. In summary, knees progress to adduction during the swing phase, flexion during the stance phase, and external tibial rotation throughout the gait cycle. There are significant differences between right and left knees. Factors associated with knee kinematics include age, sex, and BMI. However, further comprehensive studies on knee kinetics and spatio-temporal parameter are recommended to thoroughly understand the knee mechanism in Thai people.

### Electronic supplementary material

Below is the link to the electronic supplementary material.

Additional file 1

## Data Availability

The data used to support the findings of this study are available from the corresponding author upon reasonable request.
