# Supplementary material for: The knee kinematic patterns and associated factors in healthy Thai adults
Source: BMC Musculoskelet Disord. 2023 Dec 5;24:940. doi: 10.1186/s12891-023-07081-7 (PMC10696785; doi:10.1186/s12891-023-07081-7)
Supplement: Supplementary file 1 — Additional file 1 [file 12891_2023_7081_MOESM1_ESM.docx]

**Additional file 1**

**Table S1** Multiple regression model of terminal stance in the frontal plane (both knees)

| Variable | Coefficient | Standard error | 95% CI | P-value |
| --- | --- | --- | --- | --- |
| Age | -0.01 | 0.06 | -0.12, 0.09 | 0.819 |
| Female sex | -2.61 | 0.69 | -3.97, -1.25 | <0.001* |
| BMI | -0.63 | 0.16 | -0.95, -0.29 | <0.001* |
| Constant | 14.87 | 3.42 | 8.09, 21.66 | <0.001 |

Adjusted R-squared = 0.1798, P-value of F test = 0.0001

*Significant (P<0.05), CI = Confidence interval

**Table S2** Multiple regression model of terminal stance in the frontal plane (right knee)

| Variable | Coefficient | Standard error | 95% CI | P-value |
| --- | --- | --- | --- | --- |
| Age | -0.02 | 0.06 | -0.14, 0.11 | 0.808 |
| Female sex | -2.39 | 0.76 | -3.90, -0.88 | 0.002* |
| BMI | -0.68 | 0.18 | -1.05, -0.32 | <0.001* |
| Constant | 16.12 | 3.78 | 8.61, 23.63 | <0.001 |

Adjusted R-squared =0.1793, P-value of F test = 0.0003

*Significant (P<0.05), CI = Confidence interval

**Table S3** Multiple regression model of terminal stance in the frontal plane (left knee)

| Variable | Coefficient | Standard error | 95% CI | P-value |
| --- | --- | --- | --- | --- |
| Age | -0.01 | 0.06 | -0.13, 0.11 | 0.862 |
| Female sex | -2.83 | 0.74 | -4.30, -1.36 | <0.001* |
| BMI | -0.57 | 0.18 | -0.92, -0.21 | 0.002* |
| Constant | 13.63 | 3.69 | 6.30, 20.95 | <0.001 |

Adjusted R-squared =0.1565, P-value of F-test =0.0003

*Significant (P<0.05), CI = Confidence interval

**Table S4** Multiple regression model of mid-swing in the transverse plane (both knees)

| Variable | Coefficient | Standard error | 95% CI | P-value |
| --- | --- | --- | --- | --- |
| Age | -0.25 | 0.18 | -0.61, 0.10 | 0.160 |
| Female sex | -6.07 | 2.18 | -10.39, -1.73 | 0.007* |
| BMI | 0.32 | 0.52 | -0.72, 1.36 | 0.545 |
| Constant | -27.05 | 10.87 | -48.64, -5.46 | 0.015 |

Adjusted R-squared = 0.1493, P-value of F-test = 0.0004

*Significant (P<0.05), CI = Confidence interval

**Table S5** Multiple regression model of mid-swing in the transverse plane (right knee)

| Variable | Coefficient | Standard error | 95% CI | P-value |
| --- | --- | --- | --- | --- |
| Age | -0.35 | 0.21 | -0.78, 0.07 | 0.100 |
| Female sex | -5.55 | 2.61 | -10.73, -0.37 | 0.036* |
| BMI | 0.64 | 0.63 | -0.60, 1.89 | 0.308 |
| Constant | -31.93 | 12.99 | -57.74, -6.13 | 0.016 |

Adjusted R-squared =0.1529, P-value of F-test =0.0013

*Significant (P<0.05), CI = Confidence interval

**Table S6** Multiple regression model of mid-swing in the transverse plane (left knee)

| Variable | Coefficient | Standard error | 95% CI | P-value |
| --- | --- | --- | --- | --- |
| Age | -0.15 | 0.19 | -0.52, 0.22 | 0.423 |
| Female sex | -6.58 | 2.30 | -11.15, -2.01 | 0.005* |
| BMI | -0.01 | 0.55 | -1.10, 1.09 | 0.992 |
| Constant | -22.17 | 11.46 | -44.91, 0.58 | 0.056 |

Adjusted R-squared =0.1396, P-value of F-test =0.0026

*Significant (P<0.05), CI = Confidence interval
